# Unleashing Nature’s Allies: Comparing the Vertical Transmission Dynamics of Insect-Specific and Vertebrate-Infecting Flaviviruses in Mosquitoes

**DOI:** 10.3390/v16091499

**Published:** 2024-09-23

**Authors:** Alyssa J. Peterson, Roy A. Hall, Jessica J. Harrison, Jody Hobson-Peters, Leon E. Hugo

**Affiliations:** 1 Mosquito Control Laboratory, QIMR Berghofer Medical Research Institute, Herston, QLD 4006, Australia; alyssa.peterson@qimrberghofer.edu.au; 2 School of Chemistry and Molecular Biosciences, The University of Queensland, St. Lucia, QLD 4072, Australia; roy.hall@uq.edu.au (R.A.H.); j.harrison1@uq.edu.au (J.J.H.); j.peters2@uq.edu.au (J.H.-P.); 3 Australian Infectious Diseases Research Centre, Brisbane, QLD 4072, Australia; 4 School of Biomedical Sciences, The University of Queensland, St. Lucia, QLD 4072, Australia

**Keywords:** insect-specific virus, flavivirus, vertical transmission, arbovirus, biological control, transovarial transmission

## Abstract

Insect-specific viruses (ISVs) include viruses that are restricted to the infection of mosquitoes and are spread mostly through transovarial transmission. Despite using a distinct mode of transmission, ISVs are often phylogenetically related to arthropod-borne viruses (arboviruses) that are responsible for human diseases and able to infect both mosquitoes and vertebrates. ISVs can also induce a phenomenon called “superinfection exclusion”, whereby a primary ISV infection in an insect inhibits subsequent viral infections of the insect. This has sparked interest in the use of ISVs for the control of pathogenic arboviruses transmitted by mosquitoes. In particular, insect-specific flaviviruses (ISFs) have been shown to inhibit infection of vertebrate-infecting flaviviruses (VIFs) both in vitro and in vivo. This has shown potential as a new and ecologically friendly biological approach to the control of arboviral disease. For this intervention to have lasting impacts for biological control, it is imperative that ISFs are maintained in mosquito populations with high rates of vertical transmission. Therefore, these strategies will need to optimise vertical transmission of ISFs in order to establish persistently infected mosquito lines for sustainable arbovirus control. This review compares recent observations of vertical transmission of arboviral and insect-specific flaviviruses and potential determinants of transovarial transmission rates to understand how the vertical transmission of ISFs may be optimised for effective arboviral control.

## 1. Introduction

Arthropod-borne viruses, or arboviruses, are significant contributors to diseases of both medical and veterinary concern. Millions of cases of mosquito-borne viral infections occur every year, with over 390 million human infections by dengue virus (DENV) alone [1,2]. Arboviruses are primarily transmitted by mosquitoes, with transmission predominantly concentrated in tropical and subtropical regions [1,3,4]. However, in recent times, arboviruses that were historically localised to tropical areas have undergone ecological expansion due to urbanisation and global warming, leading to substantial outbreaks in more temperate regions, including West Nile virus (WNV), chikungunya virus (CHIKV), and Usutu virus outbreaks in Europe [5,6] and the outbreak of Japanese encephalitis virus (JEV) in temperate regions of Australia [7]. One of the biggest contributors to the global arbovirus burden is an increase in the global distribution of *Aedes aegypti* and *Aedes albopictus* mosquitoes, the main vectors of dengue (DENV), Zika (ZIKV), and yellow fever (YFV) viruses, due to climate change [8].

Approved vaccines are lacking for the majority of arboviral diseases, with mosquito management strategies being the primary means of disease control. While effective vaccines are available for YFV, JEV, and most recently CHIKV [9], vaccine development for DENV and ZIKV has been challenged by the complexities of the immune response [10]. The Dengvaxia vaccine (Sanofi Pasteur, Lyon, France) is only approved for dengue immune recipients, while the TAK-003/Qdenga (Takeda GmbH, Singen, Germany) vaccine has demonstrated greater efficacy and received limited regional approval [11]. The World Health Organisation launched the Global Arbovirus Initiative in March 2022 to design a multi-faceted and comprehensive plan to mitigate the spread of arboviruses with a focus on prevention, preparedness, and vector control [12]. A major challenge besetting vector control approaches includes insecticide resistance due to the widespread and indiscriminate use of insecticides [13,14,15].

An alternative approach to controlling the spread of arboviruses is through biological control, or biocontrol, the utilisation of biological resources to combat disease and pests. A precedent for the biocontrol of arboviral disease is the release of *Wolbachia* intracellular bacteria into mosquito populations to control dengue. *Wolbachia* alphaproteobacteria are naturally found in insects and nematodes and are maternally transmitted to offspring. *Wolbachia* can induce cytoplasmic incompatibility, leading to embryo lethality in crosses of infected males with uninfected females, which increases the proportion of infected individuals over time. *Ae. aegypti* mosquitoes, the primary vectors of YFV, DENV, and ZIKV, are naturally uninfected with *Wolbachia.* Microinjection of *Ae. aegypti* embryos with *Wolbachia* has produced stably infected lines that are resistant to subsequent infection of the mosquitoes with these arboviruses [16,17,18]. *Wolbachia* infections have been established in local *Ae. aegypti* populations in 11 countries by the World Mosquito Program (WMP) [19]. A randomised cluster trial in Indonesia showed that *Wolbachia* deployment was associated with a 77.1% protective efficacy against human DENV infection and an 86.2% protective efficacy against hospitalisation [20]. A different study conducted three years after *Wolbachia* infected *Ae. aegypti* were released in Niterói, Brazil, showed that *Wolbachia* had reduced dengue incidence by 69%, chikungunya incidence by 56%, and Zika incidence by 37% [21]. *Wolbachia* are model biological control agents for the arboviruses transmitted by *Ae. aegypti*, but *Wolbachia* infections have not been established in mosquito species that transmit several other important human pathogens [22].

**Figure 1:**
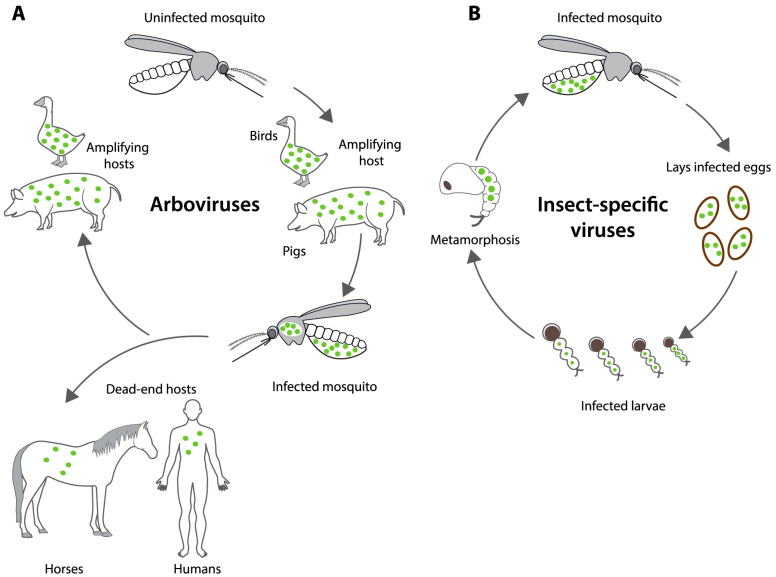
Differences in transmission of arboviruses and insect-specific viruses. (**A**). Cycle of arbovirus transmission, with an uninfected mosquito feeding on an amplifying host and spreading the virus to other vertebrate hosts. Some host species may develop sufficient viraemia to infect feeding mosquitoes, referred to as amplifying hosts, while other host species do not develop viremias that are sufficient to infect mosquitoes and are referred to as “dead end hosts”. (**B**). Vertical transmission of insect-specific viruses within mosquito populations showing the transmission of the virus directly from infected mother to offspring, without the need for virus amplification in vertebrate hosts. Green dots represent viral infection.

In the past two decades, a novel group of viruses that exclusively replicate within insect cells has come to light, referred to as insect-specific viruses (ISVs). These viruses are sometimes referred to as mosquito-specific viruses when their replication and transmission are restricted to mosquitoes; however, for the sake of this review, we will utilise the more common term of ISVs. ISVs occur within virus families that also include arbovirus pathogens of humans, including the Flaviviridae (which includes DENV, ZIKV, and YFV) and Togaviridae (which includes CHIKV), and they have been isolated from various mosquito vectors [23,24]. While most ISVs can grow efficiently in mosquito cell lines, their restriction to insects has been demonstrated by their inability to replicate in a range of vertebrate cell lines, including those derived from human, monkey, rodent, porcine, avian, and reptilian tissue [25,26,27,28,29,30,31,32,33,34]. Unlike arboviruses, which are primarily transmitted in cycles that involve alternate transmission between vertebrates and mosquito vectors, ISVs can propagate entirely within mosquito populations via vertical transmission (VT), the transmission of virus from infected females to their progeny (Figure 1) [35,36]. ISVs can also induce a phenomenon termed “superinfection exclusion”, in which a primary viral infection inhibits a secondary viral infection of a cell. Of particular interest is the capacity for certain ISVs to suppress the infection of mosquito cells by human arboviral pathogens, both in vivo and in vitro [26,37,38,39,40], raising the potential for ISVs to be employed as alternative biocontrol agents against arboviral diseases.

Arboviruses and ISVs belonging to the Flaviviridae comprise the largest proportion of the mosquito virome [41]. Given that superinfection exclusion is more likely to occur between homologous viruses [42], it is important to understand the interactions and transmission dynamics of insect-specific flaviviruses (ISFs) and flaviviral arboviruses to develop ISV-based control strategies against flaviviral pathogens.

## 2. Insect-Specific Flaviviruses (ISFs)

**Figure 2:**
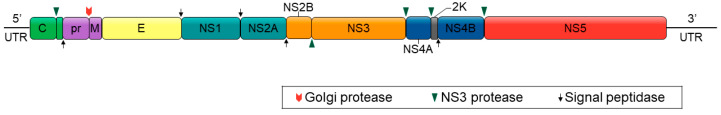
Flavivirus genome structure. Flaviviruses have a genome of approximately 11 kbp, with a single open reading frame (ORF) coding for seven non-structural proteins and three structural proteins. The polyprotein ORF is flanked on either side by 5′ and 3′ untranslated regions (UTRs) and is cleaved post-translationally by host and viral proteases. Image adapted from [55].

**Figure 3:**
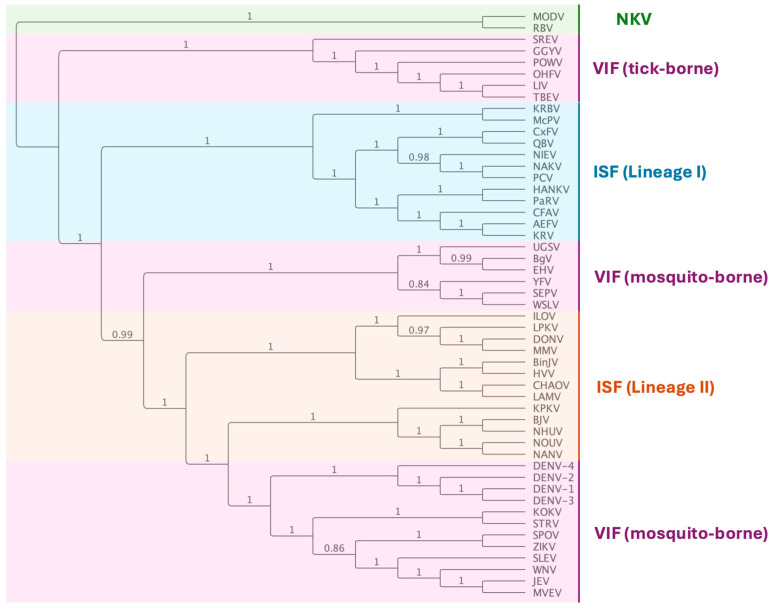
Flavivirus phylogenic tree created using maximum likelihood analysis of the complete polyprotein amino acid sequence. The tree was constructed using the methods described in [45], applying a Whelan and Goldman [56] evolutionary model optimised for Gamma likelihood with 1000 bootstrap iterations and using NKV as the outgroup. Sequences were derived from Genbank accession numbers: AB488408, *Aedes* flavivirus (AEFV); AY898809, Alfuy virus (ALFV); KU308380, Bamaga virus (BgV); KC496020, Barkedji virus (BJV); MG587038, Binjari virus (BinJV); KJ741267, cell fusing agent virus (CFAV); JQ308185, Chaoyang virus (CHAOV), AB262759, *Culex* flavivirus (CxFV); HE574574, Culex theileri flavivirus (CTFV); U88536, dengue virus serotype 1 (DENV-1); U87411, dengue virus serotype 2 (DENV-2); AY099336, dengue virus serotype 3 (DENV-3); AF326825, dengue virus serotype 4 (DENV-4); NC_016997, Donggang virus (DONV); DQ859060, Edge Hill virus (EHV); DQ235145, Gadgets Gully virus (GGYV); NC_030401, Hanko virus (HANKV); MN954647, Hidden Valley virus (HVV), KC692067, Ilomantsi virus (ILOV), AF217620, Japanese encephalitis virus (JEV), AY149905, Kamiti River virus (KRV); KY320648, Kampung Karu virus (KPKV); NC_035118, Karumba virus (KRBV); AY632541, Kokoberra virus (KOKV), KC692068, Lammi virus (LAMV); Y07863, Louping ill virus (LIV), KY290256, Long Pine Key virus (LPKV); NC_035187, Mac Peak virus (McPV); MF139576, Marisma mosquito virus (MMV); AJ242984, Modoc virus (MODV); AF161266, Murray Valley encephalitis virus (MVEV); NC_030400, Nakiwogo virus (NAKV), MF139575, Nanay virus (NANV); KJ210048, Nhumirim virus (NHUV); JQ957875, Nienokoue virus (NIEV); EU159426, Nounane virus (NOUV); AY193805, Omsk haemorrhagic fever virus (OHFV); KC505248, Palm Creek virus (PCV); KT192549, Parramatta River virus (PaRV); L06436, Powassan virus (POWV); FJ644291, Quang Binh virus (QBV); NC_003675, Rio Bravo virus (RBV); DQ235150, Saumarez Reef virus (SREV); DQ837642, Sepik virus (SEPV); KM225263, Stratford virus (STRV); DQ859064, Spondeweni virus (SPOV); DQ525916, St Louis encephalitis virus (SLEV); U27495, tick-borne encephalitis virus (TBEV); DQ859065, Uganda S virus (UGSV); JN226796, Wesselsbron virus (WSLV); KY229074, West Nile virus (WNV); MN1062241, yellow fever virus (YFV); AY632535, Zika virus (ZIKV).

Flaviviruses are single-stranded viruses with positive sense RNA genomes of approximately 11 kb [43]. The genomes of flaviviruses traditionally have a 5′ untranslated region of ~100 nt, a single open reading frame (ORF), and a 3′ untranslated region (3′ UTR) of 400–700 nt. The ORF encodes for a large polyprotein that once cleaved generates three structural proteins (capsid (C), pre-membrane/membrane (prM), and envelope (E)) and seven non-structural proteins in the order: 5′–C–prM(M)–E–NS1–NS2A–NS2B–NS3–NS4A–NS4B–NS5-3′ [44] (Figure 2). The flaviviruses can be divided into three phylogenetic groups: vertebrate-infecting viruses (VIFs) (flaviviruses that are also arboviruses), no-known vector (NKV) flaviviruses, and the ISFs [23] (Figure 3). The ISFs can be further divided into two distinct phylogenetic groups—lineage I (classical or cISFs) and lineage II (dual-host associated or dISFs), which are more closely related to VIFs [23,25,45].

The first lineage I ISF discovered, cell fusing agent virus (CFAV), was identified in a persistently infected Aag2 mosquito cell culture [46]. The subsequent detection of Kamiti River virus (KRV) in wild-caught *Aedes macintoshi* mosquitoes in Kenya [47,48] initiated the discovery of ISFs through interrogation of natural mosquito microbiomes, which has been further propelled through the advancements of deep sequencing technologies. Indeed, such discoveries have driven research into the consequences of persistent infection of ISFs (and other ISVs) in mosquito populations on the transmission dynamics of arboviral pathogens [26,38,49,50]. Such studies have revealed the ability of ISFs to exhibit superinfection exclusion of VIFs, including DENV, ZIKV, and WNV [25,51,52,53,54]. Initial studies reported by Bolling et al. [50] showed that *Culex pipiens* mosquitoes naturally infected with *Culex* flavivirus (CxFV) exhibited a significant delay in the transmission of WNV compared to controls. Subsequently, intrathoracic injection of *Culex annulirostris* mosquitoes with Palm Creek virus (PCV) reduced the infection of mosquitoes by the Kunjin strain of West Nile virus (WNV_KUN_) by 25% when compared to control (ISF-free) mosquitoes [40]. Further, titres of DENV-1 in peripheral tissues of *Ae. albopictus* were significantly lower when CFAV was injected into the mosquitoes six days before an infectious blood meal compared to mosquitoes injected two days before infection [54].

Lineage II ISFs, or dual-host-affiliated ISFs, are also unable to replicate in vertebrate cells, but their phylogenetic position places them more closely to the VIFs [25]. This may indicate that they have evolved from vertebrate-infecting viruses and have adopted an insect-specific phenotype [23]. Despite the genetic and antigenic similarity of Lineage II ISFs to VIFs, they share a similar host restriction phenotype to Lineage I ISFs, with restriction occurring at multiple levels pre- and post-entry [45]. Unsurprisingly, the comparably fewer studies that have been performed for the Lineage II ISFs show similar trends of superinfection exclusion of VIFs in vivo. Co-inoculation of *Culex quinquefasciatus* mosquitoes with WNV and Nhumirim virus (NHUV) demonstrated replication inhibition that resulted in a 40% reduction in the ability of NHUV-infected mosquitoes to transmit WNV [57].

## 3. Vertical Transmission of Flaviviruses in Mosquitoes

**Figure 4:**
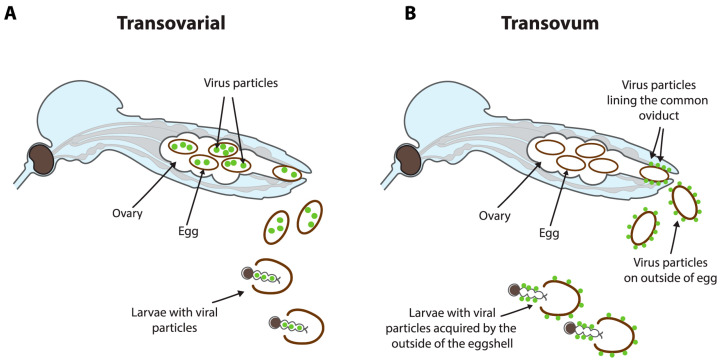
Different mechanisms of vertical transmission in mosquitoes. (**A**) Transovarial transmission occurs in the ovaries during development, where the eggs become infected with the virus internally. (**B**) Transovum transmission occurs when the eggs are infected through an infected common oviduct, in which larvae become infected after hatching. Green dots represent viral infection.

Vertical transmission is a secondary route of transmission of VIFs but is considered to be the main method of transmission for the ISFs. VT can occur by one of two routes: transovarial transmission or transovum transmission [58] (Figure 4). Transovarial transmission involves the transmission of virus from infected female mosquitoes to their developing eggs while within the ovaries [59]. Transovum transmission involves the infection of the egg during oviposition [60,61]. Infection occurs as the eggs exit the infected common oviduct during oviposition, and the virus infects the outer layers of the egg, thereby transmitting the virus from the outside of the eggshell onto the larvae after hatching [60].

Vertical transmission is usually characterised by two properties: the vertical transmission rate, defined as the proportion of infected females that transmit virus to any of their offspring, and the filial infection rate (FIR), defined as the proportion of infected larvae among all progeny from an infected mother. In field studies, however, testing for VT requires testing immature or male adult mosquitoes, for which it is impossible to know the infection status of the mother. The prevalence of immature mosquitoes that are positive for virus is often low, particularly for the VIFs (<1%), and therefore, pools of adult mosquitoes are tested to save resources. When pools of mosquitoes are tested, VT is quoted as the minimum filial infection rate (MFIR), which is calculated as the number of positive pools per total number of mosquitoes and expressed as a rate per 1000 offspring.

Understanding the differences in VT efficiency between the VIFs and ISVs and the underlying mechanisms may illuminate factors defining flavivirus host restriction and the suitability of ISVs for biocontrol. The VT of arboviruses, including VIFs, has been extensively reviewed [62,63,64,65,66]. Here, we contrast observations of natural VT between VIFs and ISFs. For the VIFs, we focus on studies occurring since the last review of arbovirus VT [64]. We also compare observations of natural VT occurring in the field and experimentally induced VT, in which the female mosquito was artificially infected with the virus.

### 3.1. Natural Occurrence of VIF and ISF Vertical Transmission in Mosquitoes

The natural occurrence of VT of flaviviruses has been demonstrated by virus detection in field-collected mosquitoes across several continents (Table 1). In these studies, VT is determined by the detection of virus in mosquitoes that have been collected in their immature stages, either as eggs, larvae, or pupae, or in male mosquitoes that do not feed on blood. Virus detections were made directly from immature stages (larvae or pupae) that were brought to the laboratory and reared to the adult stage before being tested for the presence of virus. The presence of virus was most frequently tested using variations of the reverse transcription polymerase chain reaction (RT-PCR) that detects viral genomic RNA, though some detections were made using tissue culture-based assays for virus isolation.

Sixteen investigations of the natural VT rates of VIFs since 2018 were performed on different serotypes of DENV and ZIKV on collections of up to 21,390 immature mosquitoes (Table 1). The MFIR reported for DENV ranged from 0 for DENV-1 in *Ae. aegypti* from Brazil [67] to 22.3 per 1000 for DENV-3 in *Ae. aegypti* from Mexico [63]. MFIRs for Zika virus (ZIKV) ranged from 0.5 per 1000 *Ae. aegypti* progeny from Amazonas, Brazil [58], to 7.2 per 1000 *Ae. albopictus* progeny from Mato Grosso, Brazil [68]. While many VIFs have been found to be vertically transmitted, mathematical models have shown that VT rates need to exceed 20% in order to have any impact on DENV endemicity [69]. Therefore, field observations of MIRs produced by VIFs indicate that VT is not likely to be sufficient to sustain viral presence alone.

**Table 1:** Naturally occurring flavivirus vertical transmission rates in mosquito populations.

Virus ^a^	Mosquito	Strain	Design ^b^	Detected ^c^	FIR ^d^ /100	MFIR /1000	N	Reference
***Vertebrate-infecting flaviviruses***								
DENV(1–4)	*Ae. aegypti*	Havana, Cuba	L	qRT		9.0	4102	[70]
DENV-1	*Ae. aegypti*	Sao Paulo, Brazil	L	qRT		0.0	910	[67]
DENV-1	*Ae. aegypti*	Mexico	A	nRT		0.1	2 390	[71]
DENV-2	*Ae. aegypti*	Sinaloa, Mexico	L	RT		6.5	308	[72]
DENV-2	*Ae. aegypti*	Mexico	A	nRT		1.9	2 390	[71]
DENV-3	*Ae. aegypti*	Mexico	A	nRT		2.5	2 390	[71]
DENV-3	*Ae. albopictus*	Sao Paulo, Brazil	A	qRT		0.6	3270	[64]
DENV-4	*Ae. aegypti*	Mato Grosso, Brazil	L	qRT		2.1	4490	[68]
DENV-4	*Ae. albopictus*	Mato Grosso, Brazil	L	qRT		7.0	296	[68]
DENV-4	*Ae. aegypti*	Sinaloa, Mexico	A	RT		22.3	672	[73]
DENV	*Ae. aegypti*	Yucatan, Mexico	A	qRT	0.9		1278	[74]
ZIKV	*Ae. aegypti*	Amazonas, Brazil	L	qRT		0.5	2057	[58]
ZIKV	*Ae. aegypti*	Mato Grosso, Brazil	L	qRT		1.8	4490	[68]
ZIKV	*Ae. albopictus*	Mato Grosso, Brazil	L	qRT		7.2	296	[68]
ZIKV	*Ae. aegypti*	Morelos, Mexico	L	qRT		3.9	4300	[75]
ZIKV	*Ae. aegypti*	Yucatan, Mexico	A	qRT	0.1		1278	[74]
***Lineage I Insect-specific flaviviruses***								
AeFV	*Ae. luteocephalus*	West Kenya	A	qRT		44.1	68	[76]
AeFV	*Ae. albopictus*	Bangkok	C	RT	100		130	[77]
An(g)FV	*An. gambiae*	West Kenya	A	qRT		85.7	35	[76]
CFAV	*Ae. aegypti*	West Kenya	A	qRT		4.1	729	[76]
CFAV	*Ae. aegypti*	Galveston	C	RT	100		56	[78]
CFAV	*Ae. aegypti*	Galveston ♀ × Iquitos ♂	C	RT	92.6		68	[78]
CFAV	*Ae. aegypti*	Galveston ♂ × Iquitos ♀	C	RT	75.9		104	[78]
CxFV	*Cx. pipiens*	Colorado	C	qRT	90.3		52	[79]
CxFV	*Cx. pipiens*	Iowa	A	qRT	97.4		540	[80]
PARV	*Ae. vigilax*	Sydney	A	TC		141.9		[39]
PARV	*Ae. vigilax*	Brisbane	A	TC		29.64		[39]

^a^ AeFV, *Aedes* flavivirus. *An(g)*FV, *Anopheles gambiae* flavivirus. DENV, dengue virus. CFV, cell-fusing agent virus. PARV, Parramatta River Virus. ZIKV, Zika virus. ^b^ Experiment design. L: progeny were collected as immature mosquitoes and tested as larvae. A: progeny were collected from the wild as eggs or immature mosquitoes and tested as adult mosquitoes. C: mosquitoes were obtained from a laboratory colony. ^c^ qRT, quantitative reverse transcription PCR. RT, reverse transcription PCR. nRT, nested reverse transcription PCR. TC, tissue culture. ^d^ FIR, filial infection rate. MFIR, minimum filial infection rate.

In contrast to the low VT rates observed for VIFs, lineage I ISFs were frequently observed to lead to substantially higher rates of VT. There have been few studies into the natural VT of CFAV, although a MFIR of 4.1 per 1000 was observed among *Ae. aegypti*. progeny in West Kenya [76]. However, the capacity of CFAV for substantial VT in nature is likely to be substantially higher, as CFAV was detected in more than 50% of pools of *Ae. aegypti* collected in Texas [81], and VT rates of 90% are observed in laboratory colonies (below). Very high rates of VT have been recorded for CxFv, for which near complete penetrance was observed for a population of *Cx. pipiens* in Iowa (97.4% of offspring infected) [80]. The Australian ISF Parramatta River virus (PaRV) was detected in *Aedes vigilax* progeny to a MFIR of 141.9/1000 larvae after field-collected mosquitoes were offered a single non-infectious blood meal [39]. In west Kenya, *Aedes* flavivirus (AeFV) was detected from *Aedes luteocephalus* at a MFIR of 44.1 per 1000 offspring and *Anopheles* flavivirus in *Anopheles gambiae* at an MFIR of 86.7 [76]. There is a high likelihood that vertical transmission plays an important role in the maintenance of another *Anopheles*-infecting lineage I flavivirus, Karumba virus (KRBV), from northern Australia, given high rates of detection in mosquito pools [82]. KRBV was detected at prevalences of 53–100% in mosquito pools from three locations across the region.

Laboratory strains of mosquitoes that were persistently infected with ISFs, in which progeny were infected via naturally occurring transovarial or transovum transmission, produced FIRs of between 66 and 100% (Table 1, experimental design C). Filial infection rates of 100% were observed from a strain of *Ae. albopictus* persistently infected with *Aedes* flavivirus (AeFV) [77] and the Galveston strain of *Ae. aegypti* that is persistently infected with CFAV [78]. The Galveston strain provided an opportunity to investigate the CFAV VT efficiency in mating crosses with uninfected *Ae. aegypti*. Crosses of Galveston strain females with the uninfected Iquitos strain of *Ae. aegypti* produced a 92.6% FIR among progeny, whereas crosses between Galveston males and Iquitos females produced a FIR of 75.9%. The latter result demonstrated CFAV can also be transmitted horizontally by venereal transmission, and that infection of females by this route also leads to efficient VT.

The natural VT of lineage II ISFs has not been specifically tested. However, Binjari virus (BinJV) was detected in one out of two hundred pools of *Aedes normanensi* mosquitoes, indicating low rates of VT [45]. In contrast, Hidden Valley virus (HVV) was detected in 60% of pools of *Aedeomyia catasticta* mosquitoes tested, indicating that VT may be the prominent means of transmission for this virus [45].

### 3.2. Experimentally Induced VIF and ISF Vertical Transmission Rates in Mosquitoes

The potential for VT can be inferred from experimental studies in which female mosquitoes are inoculated with the virus and the resulting proportion of infected progeny is determined. For the VIFs, female mosquitoes were most frequently challenged with virus orally, via artificial membrane feeding, but occasionally by intrathoracic (IT) microinjection (Table 2). The VT of ISFs was most often tested following virus challenge of the female by IT inoculation. In several instances, the recipient mosquitoes are not from the same species or genus as the species the virus was isolated from [40,57,83]. Viral detections were made predominantly from adult mosquitoes using quantitative RT-PCR or occasionally tissue culture ELISA methods. An emerging theme is that artificial inoculation of ISVs known to be vertically transmitted in nature has frequently not induced vertical transmission in offspring [84,85], although there are prominent examples where IT inoculation has successfully led to VT [86,87].

The VT prevalence of VIFs resulting from experimental inoculation of female mosquitoes (Table 2) was generally higher than the prevalence observed naturally for the same viruses (Table 1). Oral exposure of *Ae. albopictus* from Guangdong, China, to DENV-1 resulted in the highest recorded VT rate for VIFs, with 17.6% of offspring infected with the virus [88]. Oral exposure of a strain of *Ae. aegypti* from Mexico with DENV-2 resulted in an MFIR of between 0 and 34.4/1000 infected progeny [89]. Interestingly, there was no VT in progeny resulting from the first gonotrophic cycle (the cycle obtained from the first blood meal), but VT occurred from the second and third gonotrophic cycles, and successive cycles produced an increasing MFIR. This indicated that the likelihood of VT increased with the time available for the virus to disseminate through the mosquito and infect ovaries and developing eggs. Experimental oral exposure of *Ae. aegypti* and *Ae. albopictus* to ZIKV also resulted in substantially higher infection rates in progeny than has been observed from field collections (Table 2). Exposure of the *Ae. aegypti* UGAL strain to ZIKV resulted in an FIR of 14.3% of progeny [90]. Oral exposure of GUA and GT strains of *Ae. albopictus* from China to ZIKV resulted in increasing MIR over successive egg-laying cycles, reaching a mean of approximately 5–6% [91]. The GUA strain harbours the *Wolbachia w*AlbA and *w*AlbB strains that naturally infect this species, and a third mosquito strain, HC, was also transinfected with the *w*Pip *Wolbachia* strain isolated from *Cx. pipiens*. There was a decrease in ZIKV infection in mosquito ovaries as the number of *Wolbachia* strains increased, and complete blockage of vertical transmission occurred in the HC strain containing the triple infection. Adding *Wolbachia* strains therefore caused a cumulative reduction in ISV vertical transmission.

For mosquito strains not harbouring ISF infections, attempts have been made to establish new infection through experimental inoculation (Table 2). One of the most commonly applied inoculation methods was IT microinjection. Experiments with CFAV demonstrated the ability to induce VT of an uninfected strain of *Ae. aegypti* via IT microinjection [86]. The filial infection rate among the progeny of the injected Bangkok strain females was low (30.0%, F1 generation); however, the authors were able to increase the filial infection rate to 100% among the F2 generation by selective breeding. More recent attempts to induce VT in uninfected strains of *Ae. aegypti* by IT microinjection have not been successful [84,85]. In both instances, CFAV was obtained from long-term cell cultures (persistently infected Aag2 cells [84] and C6/36 cells [85]) in contrast to the study by Contreras-Gutierrez et al. [86], who utilised CFAV that had been more recently isolated from mosquitoes. Similarly, attempts to induce VT in uninfected *Cx. pipiens* by IT microinjection resulted in detectable virus in ovaries but not in the F1 offspring [80]. This was in contrast to the high rates of VT observed from naturally infected *Cx. pipiens* obtained from the field in the same study. A possible hypothesis to explain these discrepancies is that the capacity of ISFs for VT is determined by genomic factors, and these are selected against during long-term cell culture. The lineage I ISF Palm Creek virus (PCV) was obtained after four passages in C6/36 cells and injected into *Cx. annulirostris* females [39]. Infection rates of 95% or higher were observed in the injected mosquitoes; however, no PCV RNA was detected from the 1038 progeny tested.

Experimental inoculation of lineage II ISFs has produced varied results, although these viruses have notably been less studied. Inoculation of the UGAL strain of *Ae. aegypti* with the lineage II ISF Chaoyang virus (CHAOV) by IT microinjection resulted in a filial infection rate of 100% [87]. Another study injected NHUV from *Culex maxi* mosquitoes into *Cx. pipiens* and *Cx. quinquefasciatus* females [57]. From the progeny (n = 3) of one of the positive *Cx. pipiens* mosquitoes that were reared to adulthood, only one tested positive for VT of the virus.

**Table 2:** Experimentally achieved flavivirus vertical transmission rates in mosquito strains.

Virus ^a^	Mosquito	Strain	Design ^b^	Tested ^c^	Detection ^d^	FIR ^e^ /100	MFIR ^e^ /1000	*n*	Reference
*Vertebrate-infecting flaviviruses*									
DENV-1	*Ae. albopictus*	Guangdong	Or	L	qRT	17.6		154	[88]
DENV-2	*Ae. aegypti*	Mexico	Or (6)	L (GC1)	TC		0	500	[89]
DENV-2	*Ae. aegypti*	Mexico	Or (6)	L (GC2, 10 d)	TC		27.7	980	[89]
DENV-2	*Ae. aegypti*	Mexico	Or (6)	L (GC2, 21 d)	TC		34.4	1180	[89]
DENV-2	*Ae. aegypti*	Hano	Or	A	qRT	0.2		222	[92]
WNV	*Ae. vexans*	Connecticut	Or (5)	A	qRT		21.7	1389	[93]
ZIKV	*Ae. aegypti*	Manaus	Or (5)	A	qRT		43.3	600	[94]
ZIKV	*Ae. aegypti*	UGAL	Or	A	qRT	14.3		15	[90]
ZIKV	*Ae. aegypti*	Florida	Or (6)	A	qRT	10		56	[95]
ZIKV	*Ae. aegypti*	Florida	Or (6)	A	qRT	6.4		20	[95]
ZIKV	*Ae. albopictus*	Guangdong	Or (5)	L	RT		53.3	75	[96]
ZIKV	*Ae. albopictus*	Beijing	Or (8)	A	qRT		32.6	120	[97]
ZIKV	*Ae. albopictus*	GUA, GT	Or (6)	A	qRT	≈5–6		≈15	[91]
ZIKV	*Ae. albopictus*	HC	Or (6)	A	qRT	0		≈15	[91]
ZIKV	*Ae. albopictus*	Florida	Or (6)	A	qRT	6.4		20	[95]
ZIKV	*Ae. albopictus*	Catalonia	IT (8)	A	TC		50.25	559	[98]
*Lineage I Insect-specific flaviviruses*									
CFAV	*Ae. aegypti*	Bangkok	IT	A (F1)	RT	78.3	60		[86]
CFAV	*Ae. aegypti*	Bangkok	IT	A (F2)	RT	100.0	34		[86]
CFAV	*Ae. aegypti*	Mengei	IT (5)	A	qRT	0		60	[84]
CFAV	*Ae. aegypti*	Haikou	IT (7)	A	qRT	0		120	[84]
CFAV	*Ae. aegypti*	Poza Rico, Florida, Bangkok	IT (3)	L	qRT	0.05		1962	[85]
CxFV	*Cx. pipiens*	Iowa	IT	A	RT	0		950	[80]
KRV	*Ae. aegypti*	Nairobi	Or (7)	A	TC	3.9		410	[83]
PCV	*Cx. annulirostris*	Brisbane	IT (5)	A	RT	0		1038	[40]
*Lineage II Insect-specific flaviviruses*									
CHAOV	*Ae. aegypti*	UGAL	IT (2)	A	qRT	100		27	[87]
CHAOV-ZIKV	*Ae. aegypti*	UGAL	IT (2)	A	qRT	≈15		27	[87]
NHUV	*Cx. pipiens*	Florida	IT (9)	A	RT	66		3	[57]

Filial or minimum filial infection rates among offspring resulting from the artificial inoculation of the parental generation or within persistently infected laboratory colonies. ^a^ AeFV, *Aedes* flavivirus. *An(g)*FV, *Anopheles gambiae* flavivirus. DENV, dengue virus. CFAV, cell fusing agent virus. CHAOV. Chaoyang virus. CxFV. *Culex* flavivirus. NHUV, Nhumirim virus. PARV, Parramatta River Virus. PCV, Palm Creek virus. WNV, West Nile virus. ZIKV, Zika virus. ^b^ Experiment design. Or: mosquitoes challenged with ISF orally, by feeding. IT: mosquitoes inoculated with the ISF by intrathoracic microinjection. Numbers in parentheses denote the log value of the inoculum (pfu/mL or TCID50/mL). ^c^ Life-stage tested. A, adult. L, larvae. GC1, first gonotrophic cycle. GC2, second gonotrophic cycle. ^d^ qRT, quantitative reverse transcription PCR. RT, reverse transcription PCR. nRT, nested reverse transcription PCR. TC, tissue culture. ^e^ FIR, filial infection rate. MFIR, minimum filial infection rate.

## 4. Potential Determinants of ISF Vertical Transmission

### 4.1. Host Factors

The rates of VT have varied for individual flaviviruses between mosquito species, both in nature (Table 1) and following experimental inoculation (Table 2). The variation may be attributed to mosquito factors determining host restriction of arboviruses and ISVs and whether these vary between mosquito tissues. The susceptibility of a mosquito species to infection by viruses is determined by factors, including the presence of appropriate cell surface receptors and/or co-receptors required for viral attachment and cell entry and the permissiveness of the intracellular environment to viral replication for the production of new virions [34]. Host cell receptors have been identified for prominent flaviviral arboviruses [42] but are unknown for ISFs in mosquitoes. Being acquired through blood feeding, VIFs particularly require permissiveness of the mosquito midgut. Little is known of the relative importance of different mosquito tissues to ISF infection, though it is likely that the susceptibility of ovarian and follicular tissue is a requirement for VT. Interestingly, we have detected lineage I ISFs in mosquito midgut epithelia [39,40], an important site for the establishment of arbovirus infection. Further, studies with lineage II Binjari reporter virus revealed a likely salivary gland infection [99]. This raises the possibility of inhibitory interactions with arboviruses in these important niches for arbovirus infection and transmission.

Host innate immune mechanisms determine the trajectory of VIF and ISF infection, and recent studies indicate a role of innate immunity in VT. Once inside the insect cell, viruses are subject to host innate immune responses against viral infection [100,101]. The main antiviral immune responses are the innate JAK–STAT, Toll, IMD, and RNA interference (RNAi) pathways. RNAi pathways include the small interfering RNA (siRNA) and PIWI interfering (piRNA) pathways that can induce sequence-specific virus cleavage. In the siRNA pathway, dsRNA produced during viral replication is processed into 21 nt long siRNA by the RNAse III enzyme Dicer 2, which is then loaded into the RNA-induced silencing complex (RISC) that cleaves complementary viral RNA sequences. The piRNA pathway was first known for its role in the protection of the *Drosophila* genomes against transposable elements (TE) in germline tissues, involving the interaction of P-element-induced wimpy testis (PIWI) family proteins, Aubergine (Aub), and Agonaut 3 with genome-encoded 21–24 nt piRNAs to degrade TE. In mosquitoes, the pathway has expanded to target viral RNA genomes, guided by virus-derived piRNAs (vpiRNAs) [102].

Recent findings indicate the existence of an immune pathway involving piRNA and viral DNA (vDNA) in mosquito ovaries that may play a role in limiting VT [103,104]. In mosquitoes, viral RNA is reverse transcribed into viral DNA through endogenous cellular reverse transcriptase activity, likely arising from endogenous retrotransposons [105]. The viral DNA can occur as episomes or be integrated into mosquito genomes as non-retroviral endogenous viral elements (EVEs). EVEs are accumulated within the genome over many generations and are inherited as host alleles [106]. EVEs from the ISFs CFAV and Kamiti River virus (KRV) are present within the *Ae. aegypti* genome [107]. Viral DNA can prime early phases of small RNA responses against the cognate virus, including the production of virus-specific siRNA and PIWI-interacting RNA (piRNA) [105]. Recent studies have identified a role of a CFAV EVE in the suppression of CFAV viral replication in mosquito ovaries via the piRNA pathway [103,104]. Ablation of a CFAV EVE by genomic editing substantially reduced EVE-derived piRNAs and increased CFAV virus replication in ovaries.

A similar phenomenon of vertically transmitted viral immunity was recently demonstrated for alphaviruses (positive sense RNA viruses within the family Togaviridae) [103]. The progeny of *Ae. aegypti* infected with Semliki Forest virus (SFV) and Ross River virus contained no detectable “infectious” virus but did possess episomal and integrated viral DNA. The presence of episomal DNA was associated with resistance to subsequent challenge of the progeny with SFV, and the episomal DNA-associated resistance was inherited over four generations in the absence of further virus challenge. Treatment of the parent mosquitoes with the reverse transcriptase inhibitor Zidovudine (AZT) eliminated viral DNA from ovaries and installed VT of SFV in a small proportion of offspring.

### 4.2. Viral Factors

The differing rates of VT between VIFs and ISFs have raised questions about the contribution of specific regions of the flavivirus genome to VT. One way to assess the contribution of different viral genes is by swapping genes with other viruses using reverse genetics approaches to create viral chimeras and testing these in vitro. Such approaches have been applied to ISFs to determine the viral genomic elements responsible for host restriction, although most host-restriction work has been limited to various vertebrate cell lines [45,108,109,110,111]. These studies have enabled comparisons of host restriction of lineage I and II ISFs. Restriction of the lineage I ISFs PCV and Niénokoué virus (NIEV) in vertebrate cells occurs at multiple stages of cellular infection, including attachment/entry and assembly/release [108,111]. Chimeric viruses based on the lineage II flavivirus BinJV are capable of limited entry into vertebrate cells but face strong restriction of virus replication that is mediated by non-interferon immune and temperature-dependent mechanisms [45]. Recent studies using reporter virus particles have confirmed the capacity of ISFs for vertebrate cell entry and that strong restriction of replication occurs due to interactions between host proteins and the viral UTRs [109,110]. Similar molecular genetics approaches could be applied to establish the contribution of specific genomic regions to VT in mosquitoes.

ISF chimeras are now being applied to test the contribution of viral proteins to VT in vivo. One recent study tested the capacity of a lineage II ISF-VIF chimeric vaccine candidate to be vertically transmitted by mosquitoes [87]. *Aedes aegypti* mosquitoes microinjected with the wild-type CHAOV transmitted the virus to 100% of their offspring. In contrast, VT was almost completely suppressed in mosquitoes injected with the vaccine chimera, in which the antigenic structural proteins (prM-E) of CHAOV were replaced with the prM-E of ZIKV. Therefore, while the non-structural proteins are important for host restriction, it appears that structural proteins may have a greater role in VT for some ISFs.

### 4.3. Microbiome

Mosquito microbiota also contribute to the likelihood of ISF VT. The suppression of ZIKV VT by *Wolbachia* in the *Ae. albopictus* HC strain demonstrates the influence of microbiome in determining VT efficiency (Table 2) [91]. *Wolbachia* induced strong inhibition of CFAV in vitro [112], and interactions with other ISVs in wild mosquitoes have been reported. Virus inhibition may also be induced by other bacterial microbiota in the mosquito reproductive tract, including *Enterobacter*, *Serratia*, *Asaia* [113], and *Rosenbergiella* [114]. Interactions between these taxa and flaviviruses in mosquitoes have been reported [114,115,116]; however, it is unclear how these may affect VT. Infection of mosquitoes with pre-existing ISFs may influence the establishment of persistent infection with a desired ISF through superinfection exclusion, particularly if the endogenous virus has established a prior infection in ovaries. An appraisal of the existing bacterial and viral microbiota of a target mosquito vector by deep sequencing should be conducted before engaging in efforts to establish ISF infections.

## 5. Conclusions

Research into the VT of flaviviruses has illuminated differences between the VT of VIFs and ISFs that may guide the optimisation of sustainable ISF biocontrol strategies against arboviruses. The unique ability of certain ISFs to hinder VIF replication in mosquitoes, coupled with their exclusive infectivity to insect hosts, positions them as compelling biocontrol candidates for the control of arboviral diseases. However, attaining high VT rates will be key to achieving the sustainability of such a strategy. Observations of near-complete penetrance of mosquito populations by ISFs highlight their potential as naturally propagating biological control agents.

VT rates were consistently higher for ISFs than for VIFs, reflecting the importance of this pathway to ISF propagation. The highest rates of ISF VT were observed from select laboratory mosquito colonies that are persistently infected with ISFs. For these strains, the ISF infection was transmitted to up to 100% of the progeny. Other associations lead to incomplete penetrance of virus infection among progeny. Research on the determinants of this variation indicates that host, virus, and microbiome factors may play a role in determining the propensity of ISFs for VT. Understanding these factors is essential to optimising the VT of ISFs for biological control applications.
